# The centrality of affective instability and identity in Borderline Personality Disorder: Evidence from network analysis

**DOI:** 10.1371/journal.pone.0186695

**Published:** 2017-10-17

**Authors:** Juliette Richetin, Emanuele Preti, Giulio Costantini, Chiara De Panfilis

**Affiliations:** 1 Department of Psychology, University of Milano-Bicocca, Milano, Italy; 2 Personality Disorders Lab, Parma-Milan, Italy; 3 Department of Medicine and Surgery, University of Parma, Parma, Italy; Universita Cattolica del Sacro Cuore Sede di Roma, ITALY

## Abstract

We argue that the series of traits characterizing Borderline Personality Disorder samples do not weigh equally. In this regard, we believe that network approaches employed recently in Personality and Psychopathology research to provide information about the differential relationships among symptoms would be useful to test our claim. To our knowledge, this approach has never been applied to personality disorders. We applied network analysis to the nine Borderline Personality Disorder traits to explore their relationships in two samples drawn from university students and clinical populations (*N* = 1317 and *N* = 96, respectively). We used the Fused Graphical Lasso, a technique that allows estimating networks from different populations separately while considering their similarities and differences. Moreover, we examined centrality indices to determine the relative importance of each symptom in each network. The general structure of the two networks was very similar in the two samples, although some differences were detected. Results indicate the centrality of mainly affective instability, identity, and effort to avoid abandonment aspects in Borderline Personality Disorder. Results are consistent with the new DSM Alternative Model for Personality Disorders. We discuss them in terms of implications for therapy.

## Introduction

### Borderline Personality Disorder

Borderline Personality Disorder (BPD) is a severely impairing condition characterized by instability affecting self-image, interpersonal relationships, and affects, as well as marked impulsivity [1]. Despite the rather heated debate prompted by the last editing of the Diagnostic and Statistical Manual of Mental Disorders [1], the diagnosis of Personality Disorders (PDs) underwent only minor changes, with the 10 categorical and polythetic diagnoses still holding center stage. The pitfalls of a categorical approach to PDs have long been recognized and documented, ranging from poor discriminant validity to high comorbidity and heterogeneity of symptoms (e.g., [2]). Based on DSM criteria, a BPD diagnosis is issued if five out of a set of nine symptoms are present, so that symptoms are treated as exchangeable: Diagnosis is based on the *number* of symptoms rather than on their *specificities* [3] and does not provide indication of the specific relations among symptoms within the disorder. Nevertheless, the idea that all criteria are not “created equal” ([4], p. 886) has long been present. The criteria were presented in the DSM-IV in a descending order of diagnostic value, based on psychometric research [5]. A number of studies have then analyzed the diagnostic efficiency of the different PD criteria. Considering BPD, affective instability and, to a certain extent, identity disturbances, have been found to be particularly relevant (e.g., [6]). Moreover, clinicians rate identity disturbances and affective instability as the most causally central traits of BPD [7]. Another core feature of BPD consists in problems in interpersonal relationships. Both from a research and clinical and clinical perspective (e.g., [8], [9]), in fact, interpersonal problems, with a particular focus on abandonment concerns and intolerance of aloneness, have been found to be specific markers of BPD. It is worth noting that the dimensional alternative model for PD diagnosis of DSM-5 (AMPD DSM-5) attempts to deal with such issues by adopting a dimensional approach that posits identity and interpersonal functioning as the key defining features of PDs. Thus, it seems necessary to rely on alternative data-driven techniques capable to uncover the differential role of symptoms in the perspective of an evidence-based approach to psychiatric diagnosis [10]. In this context, the recent approach of network analysis might provide useful insights.

### The network approach for studying psychopathology

In psychiatric diagnosis, the assumption that a group of symptoms shares a causal relation with one underlying disease has been questioned [11]. Alternatively, according to the network approach, disorders are conceived as systems of connected symptoms, rather than as entities [12]. Moreover, symptoms are not interchangeable: Each one plays a unique role, which depends on its particular pattern of connections with the others.

In psychopathology networks, nodes represent symptoms and their connections (edges) represent their pairwise relationships. The interpretation of edges crucially depends on the method used for computing them. In regularized partial correlation networks estimated on cross-sectional data, typically via the Graphical Lasso algorithm [13], each edge encodes the correlation between two symptoms controlling for all others. An edge implies that the relationship is at least in part due to the two specific symptoms and cannot be attributed entirely to the others. Conversely, the absence of an edge (i.e., a zero partial correlation) means that these symptoms can be considered mutually independent [14,15]. This method has been widely employed in psychopathology (e.g., [16,17]) and it has been recently extended for simultaneously estimating networks from different populations (Fused Graphical Lasso [18,19]). Partial correlation networks do not necessarily reflect causal relations, although they can highlight potential causal pathways (see [20] for a thorough discussion about causal interpretations of network models). Furthermore, they should not be confused with graphical vector auto-regression networks (e.g., [21]), which use arrowheads to specify temporal dependencies. Partial correlation networks tell us if, for example, individuals that are often sad might also be often tired, whereas temporal networks tell us if individuals are more likely to be tired if they reported being sad the previous time they were administered the questionnaire [22].

The network approach introduces a very important concept for the understanding of psychopathology, namely centrality [23]. Centrality indices quantify several ways in which a trait or symptom plays an important role in the context provided by other symptoms. *Strength centrality* is a measure of how strongly each symptom is directly connected with the others. Strength is a very stable and widespread index of centrality (e.g., [24]). A high value implies that a symptom directly interacts with several other symptoms in the network. Including information about strength centrality in a regression model allowed improving the prediction of the onset of depression [25]. *Closeness centrality* considers all paths between a node and all other nodes in the network, including indirect connections. A change in a closeness central node is more likely to affect quickly other parts of the network and changes in any other part of the network are more likely to affect closeness central nodes. For instance, medication seems to affect closeness-central depressive symptoms more than the peripheral ones [26]. Finally, *betweenness* is the number of times a symptom lies on the shortest path between two other symptoms. Higher values reflect a symptom’s greater centrality to the network. However, betweenness is less stable than other centrality indices and thus more affected by small changes in the network estimates [27].

Recent work has demonstrated the usefulness of the network approach for understanding the differential role of symptoms in a specific disorder (e.g., [16,17,28,29]). For example, Robinaugh and colleagues [29] provide results supporting the idea that Persistent Complex Bereavement Disorder (PCBD) constitutes a causal system of mutually reinforcing symptoms that arise following the death of a loved one and not because they are caused by a common underlying disorder. The network approach highlights the importance of the overlap of symptoms because of their ability to influence both PCBD and depression networks and increase the likelihood of experiencing one when experiencing the other. Moreover, their results suggest the centrality of emotional pain in the PCBD network, contrasting with some suggestions to exclude this symptom from the diagnostic criteria (e.g., [30]). Isvoranu et al. [16] used network analysis to explore the connections between childhood trauma and psychotic symptoms. Their results showed that general psychopathology symptoms, such as anxiety and impulsivity, play a fundamental role in mediating the effects of trauma on psychosis. A final example [17] shows that hypervigilance and future foreshortening are central symptoms in the network structure of Posttraumatic Stress Disorder. The analysis also highlights differential relations between symptoms such that not all symptoms appear to form a unique and single cluster as suggested by the DSM-5. These findings demonstrate the usefulness of considering the centrality of symptoms within different disorders at least at two levels. Considering cross-sectional data, the fact that some symptoms are more central than others may help identifying the core features of the disorder, thus providing relevant information from a diagnostic point of view. Considering longitudinal data, centrality and changes in symptoms relations might help elucidating which symptoms can act as triggers for other symptoms, shedding light on developmental and maintenance mechanisms.

### Aims of the contribution

The network analysis is an insightful approach to psychopathology research from the conceptualization, diagnostic, and intervention perspectives concerning a specific disorder. However, to our knowledge, this approach has not been applied yet to personality disorders (for network analysis on normal personality see for example [31,32]). We intend to fill this gap by focusing on BPD traits. In particular, in the context of the recent interest on relations among symptoms [11], network analysis appears to be a promising tool to explore the relationships between the 9 BPD traits of the DSM. Information on trait-by-trait interactions would result in advancements both in research and clinical (i.e., assessment and treatment) approaches to BPD. Moreover, network analysis allows investigating objectively whether some traits are more central. In particular, since identity disturbances and emotion regulation are considered crucial from a diagnostic (AMPD DSM-5), theoretical (e.g., [33]), and empirical perspective [34], we expect they would cover the most central positions within the network. To achieve this aim, we estimated networks on data from a student population and a clinical sample of patients with BPD. Using network analysis, we examined the relationships between the 9 BPD criteria. Moreover, to quantify the importance of each of the 9 criteria to the BPD network, we considered three indices of centrality: Strength, closeness, and betweenness.

## Method

### Participants

For the student sample, one thousand three hundred and seventeen university students (972 women, 342 men, 3 missing data, *M* age = 22.56, range: 17–65, *SD* = 4.05) were recruited. For the clinical sample, ninety-six patients (57 women, 38 men, 1 missing data, *M* age = 37.75, range: 18–66, *SD* = 10.59) were recruited from a residential treatment facility, from a public mental health center, and at private practitioners’ offices. Inclusion criteria were age (between 18 and 75 years), presence of at least one personality disorder, absence of cognitive impairment, and no current manic episode or psychotic disorder. Data on clinical and personality disorders were gathered from clinical records. Diagnoses were attributed to patients admitted to the treatment facilities or to the private practitioners’ treatment through unstructured DSM-oriented clinical assessment conducted by a psychiatrist. Eighty-four participants (87.5%) reported one or more psychiatric diagnoses (Mood disorders, *n* = 35, 36.5%; Substance related disorders, *n* = 34, 35.4%; eating disorders, *n* = 14, 14.6%; anxiety disorders, *n* = 8, 8.3%; and other, *n* = 3, 3.1%). Thirty patients (31.2%) had more than one PD (prevalence rates of PDs are reported in Table 1). The two studies were run after review and approval from their respective university ethics committee (Milano-Bicocca and Parma). All patients and students had the adequate cognitive and language capabilities to read the information sheet and give their written informed consent to participate prior to study completion.

**Table 1:** Prevalence of Personality Disorders in the clinical sample.

	*n*	%		*n*	%
Paranoid	7	7.3	**Any Cluster B**	61	63.5
Schizoid	2	2.1	Avoidant	9	9.4
Schizotypal	3	3.1	Dependent	8	8.3
**Any Cluster A**	12	12.5	Obsessive/Compulsive	8	8.3
Antisocial	10	10.4	**Any Cluster C**	25	26
Borderline	31	32.3	Passive/Aggressive	8	8.3
Narcissistic	10	10.4	Depressive	7	7.3
Histrionic	10	10.4	NOS	32	33.3

### Procedure

All participants completed the Borderline Personality Disorder Checklist (BPDCL, [35,36]) (participants in both samples also completed other measures of personality, e.g., temperamental traits. However, given the focus on the structure and relations between the different BPD symptoms, we did not use them in the analyses and thus do not mention them further in the contribution).

### Materials

The Borderline Personality Disorder Checklist (BPDCL, [35,36]) is composed of forty-seven items that assess the severity of nine specific BPD manifestations. Respondents used 5-point Likert scales, ranging from ‘not at all’ to ‘extremely’, indicating how much they were distressed by each of 47 different experiences during the last month. The BPD Checklist’s structure is similar to the DSM-IV BPD diagnostic criteria, thus leading to the computation of a total BPD score as well as nine separate trait scores: Efforts to avoid abandonment (7 items, e.g., “*The idea of not being able to look after yourself on your own*”), unstable relationships (3 items, e.g., “*Being disappointed by someone that at first you admired or loved*”), identity disturbance (8 items, e.g., “*Uncertainty about your models and values*”), impulsivity (9 items, e.g., “*Spending impulsively too much money over your possibility*”), suicidal and para-suicidal behavior (3 items, e.g., “*The urge to commit suicide*”), affective instability (4 items, e.g., “*Feelings of despair*”), difficulty controlling anger (4 items, e.g., “*Hitting others or throwing objects toward others*), chronic feelings of emptiness (1 item, “*Feeling bored or empty inside*”), and dissociation and paranoid ideation (8 items, e.g., “*The idea of being persecuted by other people*”). Item responses were averaged for each the BPD checklist subscale and for the general score. The reliabilities ranged from .66 to .83 and from .65 to .86 for the student and the clinical samples, respectively. The clinical sample showed a significantly higher level on all nine criteria (see Table 2) (see Table in S1 Table for the descriptive statistics of the nine symptoms for men and women separately).

**Table 2:** Reliabilities and descriptive statistics of the nine BPDCL symptoms for the student and clinical samples (and difference between the two samples).

	Student (*N* = 1317)			Clinical (*N* = 96)			*t*(1411)
	α	*M*	*SD*	α	*M*	*SD*	
Efforts to avoid abandonment	.76	1.71	0.63	.75	2.19	0.83	7.05***
Unstable relationships	.71	2.13	0.95	.66	2.48	1.12	3.37***
Identity disturbance	.84	1.97	0.71	.88	2.32	0.98	4.59***
Impulsivity	.72	1.45	0.46	.65	1.72	0.60	5.44***
(Para)Suicidal behavior	.79	1.12	0.42	.78	1.82	1.07	13.54***
Affective instability	.77	2.19	0.83	.81	3.01	1.10	9.10***
Difficulty controlling anger	.66	1.61	0.62	.70	1.88	0.83	7.22***
Dissociation and paranoid ideation	.78	1.74	0.59	.80	2.09	0.82	4.02***
Chronic feelings of emptiness	—	2.47	1.15	—	3.36	1.36	5.30***

*Note*. Reliability coefficients are not reported for chronic feelings of emptiness (1 item).

*** *p* < .001.

## Results

### Network construction

**Fig 1:**
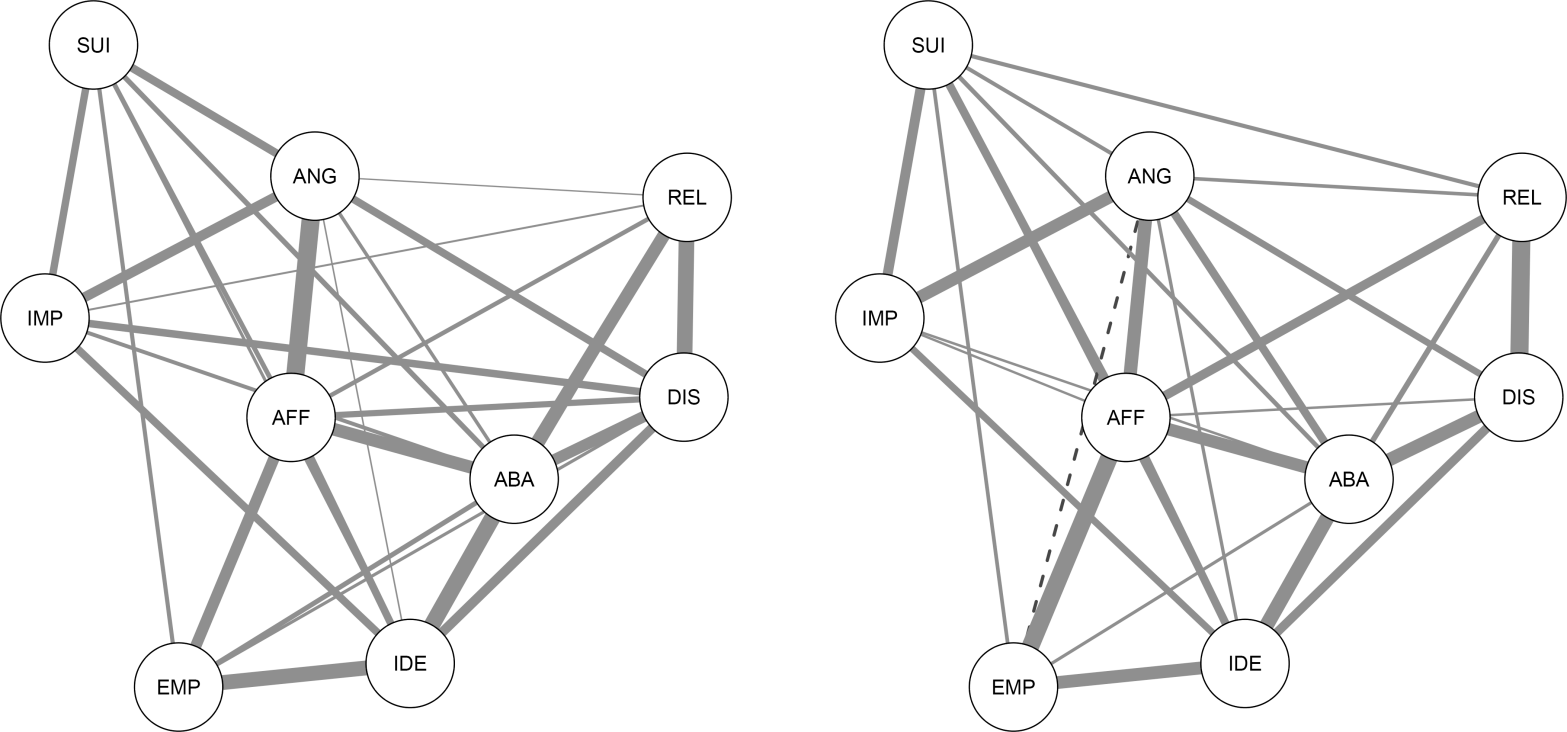
**Networks of the 9 symptoms of Borderline Personality Disorder in the student (left panel) and clinical (right panel) samples.** ABA: Efforts to avoid abandonment, REL: Unstable Relationships, IDE: Identity disturbance, IMP: Impulsivity, SUI: Suicidal and para-suicidal behavior, AFF: Affective Instability, EMP: Chronic feelings of emptiness, ANG: Difficulty controlling anger, DIS: Dissociation and paranoid ideation. Darker edges represent stronger links, full edges positive links and dashed edges negative links.

A nonparanormal transformation was applied before computing the networks, to relax the normality assumption [16] (see S1 File for dataset). Partial correlation networks were then estimated in the two samples using the Fused Graphical Lasso regularization method [18,19], as implemented in the R package *EstimateGroupNetwork* [37]. This method yields two networks, which are however estimated *jointly*. Fused Graphical Lasso improves edge estimates by exploiting similarities between samples. However, if the true networks are very different and exploiting similarities does not improve model fit, this method becomes close to estimating networks independently, therefore allowing true differences to emerge. This property makes the Fused Graphical Lasso an ideal method for estimating networks in different groups (for full details about the computation of the networks, as well as for a more detailed description of the Fused Graphical Lasso, see S2 File; for the full correlation matrices in each sample, see Table in S2 Table). This method has been used to estimate networks in cancer patients and controls [18], in different groups of PTSD patients [38], in men and women [19], and in different national samples [39]. Fig 1 reports the two networks, one for the clinical and one for the student sample. In the student and the clinical samples, 30 and 29 of all possible 36 edges (83.3% and 80.6%, respectively) were estimated to be above zero, meaning that the traits had substantial connections to each other.

Twenty-six edges were present in both networks. In terms of unique edges, the (para)suicidal behavior node is connected to unstable relationship (.060), the difficulty controlling anger node is negatively connected to chronic feelings of emptiness (-.043), and affective instability is connected to impulsivity (.024) only in the clinical sample but not in the student one. Conversely, the connection between dissociation and paranoid ideation and the nodes of impulsivity (.280) and chronic feelings of emptiness (.037), and the connection between unstable relationships and the nodes of identity disturbance (.122) and impulsivity (.014) are observed only in the student sample but not in the clinical one.

In the student sample, absolute edge values ranged from .003 (difficulty controlling anger with unstable relationship) to .328 (difficulty controlling anger with affective instability) and in the clinical sample, values ranged from .024 (impulsivity with affective instability) to .355 (unstable relationships with dissociation and paranoid ideation). Note that in both the student and the clinical sample, the edge between identity disturbance and effort to avoid abandonment (.291 and .280, respectively) and the edge between unstable relationships and dissociation and paranoid ideation (.280 and .355, respectively) are amongst the three highest values in both samples (see Table in S3 Table for all exact values).

### Centrality

**Fig 2:**
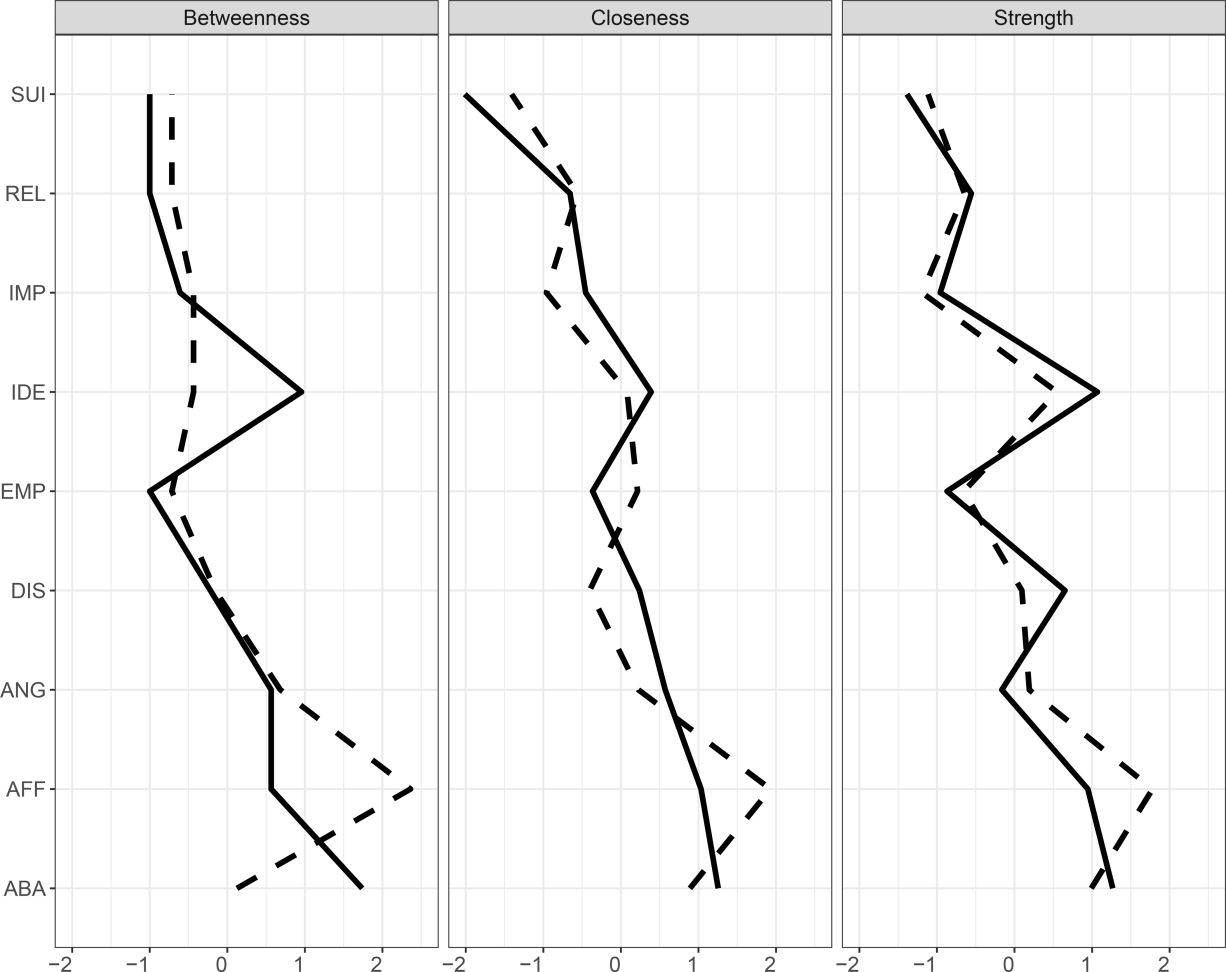
Nodes centralities for each of the nine symptoms of Borderline Personality Disorder for the student (full lines) and clinical (dashed lines) samples. ABA: Efforts to avoid abandonment, REL: Unstable Relationships, IDE: Identity disturbance, IMP: Impulsivity, SUI: Suicidal and para-suicidal behavior, AFF: Affective Instability, EMP: Chronic feelings of emptiness, ANG: Difficulty controlling anger, DIS: Dissociation and paranoid ideation. For ease of comparison for Fig 2, centrality values were standardized (z-scored) in each sample (see Table in S4 Table for exact values).

Three centrality indices, strength, closeness and betweenness, were computed using the R package *qgraph* [40]. Fig 2 illustrates each centrality index for the two samples for all nine traits (see Table in S4 Table for exact values and Table in S5 Table for the correlations between the centrality indexes indicating convergence of the results). Considering our centrality hypothesis, affective instability is the most central trait across indices in the clinical sample and among the three most central in the student sample. Identity disturbances, with the exception of betweenness in the clinical sample, is among the three most central traits. It is worth noting that effort to avoid of abandonment is also consistently among the three most central traits.

## Discussion

The network analysis approach allows providing some important insights for the 9-criteria BPD structure. We performed a network analysis on both a large student sample and a medium clinical sample with the Fused Graphical Lasso method, which improves the network estimates while preserving the specificities of the two samples. We obtained relatively robust results across the two samples. First, the two networks are in fact relatively similar with 26 common edges. Nevertheless, some edges were unique for the clinical sample, underlying a specific interplay between nodes related to severe behavioral manifestations of BPD, such as aggression and impulse control (i.e., uncontrolled anger and impulsivity), and nodes representing psychological states (emptiness) and processes (affective instability). It seems thus that these psychological states are linked with most dysfunctional behaviors only when the BPD pathology is fully exacerbated (i.e., in the clinical sample). Second, in terms of traits centrality across the two samples, affective instability is a central node of BPD, especially in the clinical sample. Identity disturbances also play a central role, although to a lesser extent. Finally, besides those two traits, effort to avoid abandonment appears to have also a central role.

The use of network analysis allows adding new information to address the issue of traits heterogeneity in the diagnosis. In this light, the consideration of the connections among traits and their different relative importance in shaping pathologies extends the results obtained from a latent disorder approach (results from a Confirmatory Factor Analysis is reported in S3 File). Network analysis starts from the assumption that traits are not interchangeable indicators (e.g., [3]). In this perspective, our results show that both in clinical and student samples, affective instability and identity disturbances play a particularly crucial role in the structure of BPD-related psychopathology. With respect to affective instability, cognitive-behavioral theories have long conceived BPD psychopathology as arising from core difficulties in emotion regulation. Linehan [41] links affective instability to the experiences of abuse and emotional invalidation often experienced by these patients, whereas other authors refer to temperamental features [42]. The central role of an altered sense of identity in personality pathology has been recently recognized in the AMPD of DSM-5 [1], which posits that moderate to extreme levels of impairment in the sense of self (i.e., stability of self-image and self-esteem, accuracy of self-appraisal, capacity for emotion tolerance and regulation) are crucial for a PD diagnosis. Notably, according to object relations theories of severe personality disorders (e.g., [43]) both identity disturbance and affective instability are defining features of BPD that result from the presence of unrealistic, partial and affectively polarized representations of self in relation to others connected to the use of primitive defense mechanisms (e.g., splitting). Such lack of integration in the sense of self, others, and corresponding affect states prevent the individual to hold a stable and continue view of self and significant others as well as to flexibly regulate one’s own affects in interpersonal realms. Considering other perspectives, the importance of identity problems in BPD has been conceptualized as a result of dissociative processes (e.g., [44]) and within the framework of attachment theory (e.g., [45,46]). Finally, the centrality of effort to avoid abandonment is consistent with the theoretical and clinical decision of having it as the first criterion of the set of BPD traits in DSM-IV [5]. Furthermore, within the AMPD of DSM 5 [1], interpersonal problems are defined as the second pillar of PD diagnosis. This is also coherent with the literature on social cognitive dysfunctions in BPD. BPD patients show increased negative affect in response to interpersonal rejection [47] and also report reduced negative emotions during social interaction only during conditions of extreme over-inclusion [48]. Moreover this is coherent with the idea that interpersonal disturbances are central in BPD (e.g.[49]) and with attachment [45,50] and mentalization research [46].

The network approach does not only add new information to the conceptualization of a pathology but could also have some implications on the way interventions might be elaborated. Although our cross sectional design does not allow drawing conclusions on the directionality [22], considering the centrality of some traits compared to others, therapeutic interventions might be more efficient targeting some specific traits and relations between traits rather than targeting the disorder [51] (for a similar reasoning on depression, see[52]). On the one hand, our results suggest that interventions could be more likely to produce change if they focus on affective instability and identity disturbances. This is consistent with the primary therapeutic focuses of two of the main treatment approaches to BPD, Dialectical-Behavioral Therapy (DBT, [41]) and Transference-Focused Psychotherapy (TFP, [53]). DBT has in fact a primary focus on the regulation of instable and extreme affective states, while identity integration (i.e., changing polarized and split self- and other-representations into a more integrated and nuanced view of self and others) is the primary therapeutic goal of TFP. The rationale of these approaches is that changing these core features will produce a change on the other traits and behavioral manifestations of the disorder. On the other hand, our results suggest that changes could be more likely detected in affective instability and identity disturbances when interventions focus on other traits. In fact, both DBT and TFP incorporate technical and tactical instruments (a supportive stance for the first, a focus on contract phase for the second) to tackle destructive behavioral manifestations (i.e., anger and suicidal behaviors). Data from randomized controlled trials (e.g., [54–57]) show that such treatments specifically reduced suicidality and anger. In particular, TFP is the only treatment that showed changes in the underlying psychological states (i.e., the nodes that only in the clinical sample are connected to severe behavioral manifestations, such as identity disturbance and chronic feelings of emptiness), specifically in reflective functioning (e.g., [58]) and identity integration [59]. These results converge with our results from the network analysis showing the centrality of the identity and affective regulation nodes. Finally our results related to the centrality of efforts to avoid abandonment highlight the importance of addressing interpersonal problems in BPD as core features with the potential to trigger other symptoms. Mentalization-Based Treatment [60], another evidence-based approach to BPD, deals with this issue by addressing disturbances in the attachment system within the context of the therapeutic relationship [61].

### Future directions

Mirroring research on depression [25], an important task for future research would examine whether the most central traits of BPD such as identity disturbance and affective instability are better than other traits in predicting the emergence of the full-blown disorder. To extend further the investigation of the role of affective instability, identity disturbance and effort to avoid abandonment, it would be important to examine the dynamic relations between the different BPD traits investigating longitudinal data in the course of therapy. Dynamic processes are indeed crucial in determining how a psychopathological condition evolves over time (see [62] for evolution of depression with a network approach) and in response to treatment. One could compare two groups before and during two different treatments targeting different traits (see [63] for a comparison of the moment-to-moment relationships between mental states) and examine, for example, whether targeting affective instability would affect connected traits, or whether affective instability would show greater progress by targeting another connected trait. Thus, approaches that combine longitudinal experimental design and network analysis [21] could provide further insights on the differential roles of the different BPD traits, thus bringing precious information for diagnosis and therapeutic intervention.

Future research could also aim to test the robustness of the solutions we obtained and their generalizability by examining the network structure of BPD criteria on a BPD only sample and other non-clinical samples using a clinician-rated measure such as a DSM-oriented clinical interview (e.g., SCID-5_PD, [64]) or dimensional interviews relying on specific theoretical models such as the Structured Interview of Personality Organization (STIPO, [65]). Besides addressing some limitations of our contribution (i.e., no BPD only sample, patients from different clinical settings, BPD diagnosis based only on symptoms experienced in the last month), this would also allow comparing networks of different groups of BPD patients to examine whether the centrality indices (i.e., the network) of traits are similar or different across different BPD “phenotypes” [66] and to address simultaneously the comorbidity or heterogeneity of traits (e.g., [67]) and traits severity issues. This would connect the network approach to previous attempts to identify different subgroups of BPD patients, that yielded sound results both empirically and in terms of clinical usefulness (e.g., [68]).

## Conclusion

To our knowledge, this contribution represents the first attempt to apply the network analysis approach to Borderline Personality Disorder traits by considering two different samples and taking into account statistically the similarities and differences between the two. This contribution provides support for the central role of affective instability, identity disturbances, and effort to avoid abandonment in Borderline Personality Disorder diagnosis.

## Supporting information

S1 File Data set. (CSV)

S2 File Network estimation. (DOCX)

S3 File Confirmatory Factor Analysis. (DOCX)

S1 Table Reliabilities and descriptive statistics of the nine BPDCL symptoms for men and women samples. (DOCX)

S2 Table Correlations among the nine symptoms of Borderline Personality Disorder in the student sample (below the diagonal) and in the clinical sample (above the diagonal). (DOCX)

S3 Table Edge values of the nine symptoms of Borderline Personality Disorder in the student (below the diagonal) and clinical (above the diagonal) samples. (DOCX)

S4 Table Centrality indices of nine symptoms of the Borderline Personality Disorder in the student and clinical samples. (DOCX)

S5 Table Correlations among different centrality measures in the student (below the diagonal) and the clinical (above the diagonal) samples and correlations between the same indices in the two samples (diagonal). (DOCX)
