# Supplementary material for: The centrality of affective instability and identity in Borderline Personality Disorder: Evidence from network analysis
Source: PLoS One. 2017 Oct 17;12(10):e0186695. doi: 10.1371/journal.pone.0186695 (PMC5645155; doi:10.1371/journal.pone.0186695)
Supplement: S2 File — (DOCX) [file pone.0186695.s002.docx]

**Supplementary Material S2**

We estimated *partial correlation networks* [1] in the student and clinical samples using the Fused Graphical Lasso (FGL; Danaher, Wang, & Witten, 2014) implemented in the R package *EstimateGroupNetwork* [3]. In the following, we introduce FGL and we provide a detailed explanation of the steps that we followed for estimating the networks, to ensure exact reproducibility of our analysis.

**Introduction to the Fused Graphical Lasso**

In psychological networks, edges typically represent estimates of partial correlations [1]. Due to sampling error, maximum likelihood (or ordinary least squares) estimates of partial correlations are never exactly zero, even when two symptoms are actually independent; furthermore estimates can be often affected by overfitting, especially with a large number of covariates [4]. Regularization through the least absolute shrinkage and selection operator (*lasso*; [5]) assuages both these issues [6], therefore methods for estimating psychological networks typically rely on this type of regularization [1,7–9]. Among lasso regularization methods, the graphical lasso is the most widespread for estimating networks on normally distributed data [1,10,11]. Instead of maximizing the log-likelihood function to yield maximum likelihood estimates, this method maximizes a *penalized* log-likelihood, a log-likelihood function plus a term that depends on network density (the number and the weights of edges). A tuning parameter (λ1) allows regulating the importance of the density penalty. Larger values of λ1 yield sparser networks (i.e., with fewer and weaker edges), whereas smaller values yield denser networks. If λ1 is set equal to zero, the penalty becomes null and the graphical lasso simply returns maximum likelihood estimates.

Sometimes it is necessary to estimate networks on the same variables measured in different classes of observations (in our case, students and patients). If the true networks were similar enough, it would be possible to improve estimates by pooling all samples together and estimating a single network. However, this strategy would also overlook any difference among classes. Conversely, estimating networks individually would allow detecting such differences, but edges would be computed separately in each sample. If the true networks were similar, using this second strategy would result in poorer estimates [2]. The FGL is a recently developed extension of the Graphical Lasso aimed at solving this problem by *jointly* estimating networks in different classes [2,12]. Like the graphical lasso, FGL includes a penalty on density, regulated by the tuning parameter λ_1_. Unlike the graphical lasso, the FGL includes also a penalty on differences among networks, regulated by a tuning parameter λ_2_. Large value of λ_2_ yield very similar networks, in which edges are estimated by exploiting all samples together. Conversely, small values of λ_2_ allow network estimates to differ. If λ_2_ is equal to zero, the networks are estimated independently of each other.

For selecting the best values of λ_1_ and λ_2_, the Extended Bayesian Information Criterion can be used (EBIC; Chen & Chen, 2008; Foygel & Drton, 2010), which performs well in simulation studies [15]. It is important to notice that FGL does not assume that the true networks are similar, because the value of λ_2_ is selected empirically according to the EBIC. If the model fit did not improve by exploiting similarities among networks, the parameter λ_2_ would be selected to be very close to zero and the FGL would nearly reduce to estimating the networks independently, without masking their differences. Conversely, if a large value of λ_2_ were selected, edges would be estimated jointly, therefore exploiting similarities across networks to improve the estimates. Intermediate values of λ_2_ allow estimating networks by both exploiting their similarities, without masking their true differences.

**Network estimation procedure**

We used the following steps for estimating the two networks:

1. To relax the normality assumption, the nonparanormal transformation implemented in the R package *huge* [16,17], was applied to the data from each class (see also [1,18]).
2. One-hundred candidate values of λ_1_ and 100 values of λ_2_ were considered. The 100 values of λ_1_ were logarithmically spaced between two values, λ_1min_ and λ_1max_. As λ_1max_ we selected the value under which no edge was retained in at least one network; λ_1min_ was defined as 0.01* λ_1max_ (see also [15]). For each value of λ_1_, 100 values of λ_2_ were selected, uniformly spaced between λ_2min_ and λ_2max_. As λ_2max_ we selected the value under which all edges were estimated to be identical in the two classes (the student and the clinical samples); λ_2min_ was defined as 0.01* λ_2max_. This tuning parameter selection strategy is implemented in function EstimateGroupNetwork within the homonymous package, by selecting setting parameter strategy = “simultaneous”.
3. Among the 10000 unique pairs of candidate λ_1_ and λ_2_ values, we selected those associated to the minimum value of EBIC, which is the default option in EstimateGroupNetwork. The values identified for the tuning parameters were λ_1_ = 0.024056083 and λ_2_ = 0.002908718 and were used for estimating the two networks. The values of the edges in the two networks are reported in Table S3, whereas centrality values are reported in Table S4.

**References**

1. Epskamp S, Fried EI. A tutorial on regularized partial correlation networks. arXiv Prepr arXiv. 2016; 1–28.

2. Danaher P, Wang P, Witten DM. The joint graphical lasso for inverse covariance estimation across multiple classes. J R Stat Soc Ser B (Statistical Methodol. 2014;76: 373–397. doi:10.1111/rssb.12033

3. Costantini G, Epskamp S. EstimateGroupNetwork: Perform the Joint Graphical Lasso and select tuning parameters. R package version 0.1.2. 2017.

4. Babyak MA. What you see may not be what you get: A brief, nontechnical introduction to overfitting in regression-type models. Psychosom Med. 2004;66: 411–421. doi:10.1097/00006842-200405000-00021

5. Tibshirani R. Regression shrinkage and selection via the lasso. J R Stat Soc B. 1996;58: 267–288.

6. McNeish DM. Using Lasso for Predictor Selection and to Assuage Overfitting: A Method Long Overlooked in Behavioral Sciences. Multivariate Behav Res. 2015;50: 471–484. doi:10.1080/00273171.2015.1036965

7. Costantini G, Epskamp S, Borsboom D, Perugini M, Mõttus R, Waldorp LJ, et al. State of the aRt personality research: A tutorial on network analysis of personality data in R. J Res Pers. 2015;54: 13–29. doi:10.1016/j.jrp.2014.07.003

8. van Borkulo CD, Borsboom D, Epskamp S, Blanken TF, Boschloo L, Schoevers RA, et al. A new method for constructing networks from binary data. Sci Rep. 2014;4: 1–10. doi:10.1038/srep05918

9. Epskamp S, Fried EI. A Tutorial on Regularized Partial Correlation Networks. Arxiv Prepr (ID 160701367). 2016; 1–21.

10. Friedman J, Hastie T, Tibshirani R. Sparse inverse covariance estimation with the graphical lasso. Biostatistics. 2008;9: 432–441. doi:10.1093/biostatistics/kxm045

11. Epskamp S, Waldorp LJ, Mõttus R, Borsboom D. Discovering psychological dynamics: The gaussian graphical model in cross-sectional and time-series data [Internet]. 2017 Sep. Available: http://arxiv.org/abs/1609.04156v3

12. Costantini G, Richetin J, Preti E, Casini E, Epskamp S, Perugini M. Stability and variability of personality networks. A tutorial on recent developments in network psychometrics. Pers Individ Dif. 2017; doi:10.1016/j.paid.2017.06.011

13. Foygel R, Drton M. Extended Bayesian information criteria for Gaussian graphical models. Adv Neural Inf Process Syst. 2010;22: 2020–2028.

14. Chen J, Chen Z. Extended Bayesian information criteria for model selection with large model spaces. Biometrika. 2008;95: 759–771. doi:10.1093/biomet/asn034

15. Epskamp S. Regularized gaussian psychological networks: Brief report on the performance of extended BIC model selection [Internet]. 2016. Available: http://arxiv.org/abs/1606.05771

16. Zhao T, Liu H, Roeder K. The huge package for high-dimensional undirected graph estimation in r. J Mach …. 2012;13: 1059–1062. Available: http://dl.acm.org/citation.cfm?id=2343681

17. Liu H, Lafferty J, Wasserman L. The nonparanormal: semiparametric estimation of high dimensional undirected graphs. J Mach Learn Res. 2009;10: 2295–2328. doi:10.1016/0006-291X(91)91267-G

18. Isvoranu A-M, van Borkulo CD, Boyette L-L, Wigman JTW, Vinkers CH, Borsboom D. A network approach to psychosis: Pathways between childhood trauma and psychotic symptoms. Schizophr Bull. 2017;43: 187–196. doi:10.1093/schbul/sbw055
