# Supplementary material for: The centrality of affective instability and identity in Borderline Personality Disorder: Evidence from network analysis
Source: PLoS One. 2017 Oct 17;12(10):e0186695. doi: 10.1371/journal.pone.0186695 (PMC5645155; doi:10.1371/journal.pone.0186695)
Supplement: S3 File — (DOCX) [file pone.0186695.s003.docx]

We fitted a CFA model using the R package lavaan (Rosseel, 2012). Since we could not assume normality, we used the Satorra-Bentler scaled χ^2^ statistic and the corresponding scaled CFI and rmsea to evaluate model fit (Finney & DiStefano, 2013). We specified a multiple-group model to examine whether a single-factor structure fitted the nine symptoms of the Borderline Personality Disorder Checklist (BPDCL, Arntz et al. 2003; Prunas et al. 2006) both in the student and in the clinical samples. This model, called *configural-invariance*, is a precondition for further assessing measurement invariance in the two samples with stronger constraints (e.g., weak, strong, and strict invariance; Hirschfeld & von Brachel, 2014; Schoot, Lugtig, & Hox, 2012). A good model fit would imply that a latent variable model in which the BPD is the common cause underlying the nine BPD symptoms, would fit the data well (although it would not automatically imply that the model is theoretically correct; Schmittmann et al., 2013). The model fit was not satisfactory, χ^2^(54) = 456.604, *p* < .001, CFI = .935, RMSEA = .103. In particular the value of the RMSEA was substantially larger than the cutoff of .06 (Hu & Bentler, 1999). We followed up this result by inspecting whether the single-factor model fitted the student or the clinical data separately. This model did not provide a good fit to the student data, χ^2^(27) = 365.167, *p* < .001, CFI = .937, RMSEA = .098, nor to the clinical data χ^2^(27) = 57.379, *p* < .001, CFI = .935, RMSEA = .108.
