# Supplementary material for: The centrality of affective instability and identity in Borderline Personality Disorder: Evidence from network analysis
Source: PLoS One. 2017 Oct 17;12(10):e0186695. doi: 10.1371/journal.pone.0186695 (PMC5645155; doi:10.1371/journal.pone.0186695)
Supplement: S1 Table — (DOCX) [file pone.0186695.s004.docx]

**Table. Descriptive statistics by gender**

|  | Men (*N* = 380) | | Women (*N* =1030 ) | |
| --- | --- | --- | --- | --- |
|  | *M* | *SD* | *M* | *SD* |
| Efforts to avoid abandonment | 1.67 | 0.62 | 1.77 | 0.67 |
| Unstable relationships | 2.06 | 0.94 | 2.19 | 0.97 |
| Identity disturbance | 1.96 | 0.72 | 2.01 | 0.74 |
| Impulsivity | 1.61 | 0.54 | 1.42 | 0.44 |
| (Para)Suicidal behavior | 1.19 | 0.55 | 1.16 | 0.51 |
| Affective instability | 2.10 | 0.84 | 2.30 | 0.88 |
| Difficulty controlling anger | 1.67 | 0.68 | 1.62 | 0.62 |
| Dissociation and paranoid ideation | 1.75 | 0.59 | 1.77 | 0.62 |
| Chronic feelings of emptiness | 2.50 | 1.21 | 2.55 | 1.18 |
