# Supplementary material for: The centrality of affective instability and identity in Borderline Personality Disorder: Evidence from network analysis
Source: PLoS One. 2017 Oct 17;12(10):e0186695. doi: 10.1371/journal.pone.0186695 (PMC5645155; doi:10.1371/journal.pone.0186695)
Supplement: S2 Table — (DOCX) [file pone.0186695.s005.docx]

**Table. Correlations among the nine symptoms of Borderline Personality Disorder in the student sample (below the diagonal) and in the clinical sample (above the diagonal)**

|  | 1 | 2 | 3 | 4 | 5 | 6 | 7 | 8 | 9 |
| --- | --- | --- | --- | --- | --- | --- | --- | --- | --- |
| 1. Effort to avoid abandonment | -- | .676 | .742 | .469 | .506 | .759 | .592 | .702 | .730 |
| 2. Unstable relationships | .667 | -- | .573 | .391 | .433 | .646 | .460 | .584 | .735 |
| 3. Identity disturbance | .751 | .636 | -- | .490 | .439 | .718 | .630 | .632 | .675 |
| 4. Impulsivity | .512 | .409 | .516 | -- | .490 | .486 | .371 | .593 | .413 |
| 5. (Para)suicidal behavior | .522 | .315 | .415 | .479 | -- | .526 | .420 | .504 | .429 |
| 6. Affective instability | .729 | .587 | .691 | .453 | .420 | -- | .736 | .674 | .641 |
| 7. Chronic feelings of emptiness | .567 | .468 | .647 | .329 | .308 | .606 | -- | .434 | .473 |
| 8. Difficulty of controlling anger | .605 | .475 | .555 | .516 | .502 | .658 | .438 | -- | .646 |
| 9. Dissociation and paranoid ideation | .742 | .680 | .715 | .536 | .444 | .672 | .535 | .604 | -- |

*Note*. All correlations *p* < .001.
