# Supplementary material for: The centrality of affective instability and identity in Borderline Personality Disorder: Evidence from network analysis
Source: PLoS One. 2017 Oct 17;12(10):e0186695. doi: 10.1371/journal.pone.0186695 (PMC5645155; doi:10.1371/journal.pone.0186695)
Supplement: S3 Table — (DOCX) [file pone.0186695.s006.docx]

**Table. Edge values of the nine symptoms of Borderline Personality Disorder in the student (below the diagonal) and clinical (above the diagonal) samples.**

|  | 1 | 2 | 3 | 4 | 5 | 6 | 7 | 8 | 9 |
| --- | --- | --- | --- | --- | --- | --- | --- | --- | --- |
| 1. Effort to avoid abandonment | -- | .089 | .280 | .027 | .068 | .235 | .040 | .144 | .252 |
| 2. Unstable relationships | .224 | -- | 0 | 0 | .060 | .167 | 0 | .049 | .355 |
| 3. Identity disturbance | .291 | .122 | -- | .118 | .036 | .142 | .243 | .043 | .162 |
| 4. Impulsivity | .052 | .014 | .124 | -- | .183 | .024 | 0 | .231 | 0 |
| 5. (Para)suicidal behavior | .077 | 0 | .035 | .120 | -- | .147 | .051 | .057 | 0 |
| 6. Affective instability | .223 | .061 | .121 | 0 | .068 | -- | .342 | .252 | .026 |
| 7. Chronic feelings of emptiness | .071 | 0 | .264 | 0 | .056 | .195 | -- | -.043 | 0 |
| 8. Difficulty of controlling anger | .043 | .003 | .006 | .169 | .134 | .328 | 0 | -- | .114 |
| 9. Dissociation and paranoid ideation | .196 | .280 | .162 | .122 | 0 | .096 | .037 | .124 | -- |
