# Supplementary material for: The centrality of affective instability and identity in Borderline Personality Disorder: Evidence from network analysis
Source: PLoS One. 2017 Oct 17;12(10):e0186695. doi: 10.1371/journal.pone.0186695 (PMC5645155; doi:10.1371/journal.pone.0186695)
Supplement: S4 Table — (DOCX) [file pone.0186695.s007.docx]

**Table. Centrality indices of nine symptoms of the Borderline Personality Disorder in the student and clinical samples.**

|  | Betweenness | | Closeness | | Strength | |
| --- | --- | --- | --- | --- | --- | --- |
|  | Student | Clinical | Student | Clinical | Student | Clinical |
| Efforts to avoid abandonment | 7 | 3 | 0.0176 | 0.0182 | 1.1769 | 1.1352 |
| Unstable relationships | 0 | 0 | 0.0129 | 0.0142 | 0.7041 | 0.7213 |
| Identity disturbance | 5 | 1 | 0.0155 | 0.0159 | 1.1258 | 1.0238 |
| Impulsivity | 1 | 1 | 0.0134 | 0.0131 | 0.6002 | 0.5830 |
| (Para)Suicidal behavior | 0 | 0 | 0.0096 | 0.0118 | 0.4889 | 0.6024 |
| Affective Instability | 4 | 11 | 0.0171 | 0.0210 | 1.0936 | 1.3354 |
| Chronic feelings of emptiness | 0 | 0 | 0.0136 | 0.0163 | 0.6231 | 0.7191 |
| Difficulty controlling Anger | 4 | 5 | 0.0159 | 0.0163 | 0.8061 | 0.9330 |
| Dissociation and paranoid ideation | 2 | 2 | 0.0151 | 0.0146 | 1.0172 | 0.9088 |
