# Supplementary material for: The centrality of affective instability and identity in Borderline Personality Disorder: Evidence from network analysis
Source: PLoS One. 2017 Oct 17;12(10):e0186695. doi: 10.1371/journal.pone.0186695 (PMC5645155; doi:10.1371/journal.pone.0186695)
Supplement: S5 Table — (DOCX) [file pone.0186695.s008.docx]

**Table. Correlations among different centrality measures in the student (below the diagonal) and the clinical (above the diagonal) samples and correlations between the same indices in the two samples (diagonal).**

|  | Strength | Closeness | Betweenness |
| --- | --- | --- | --- |
| Strength | **.90**** | .91** | .81* |
| Closeness | .87** | **.84*** | .82* |
| Betweenness | .87** | .84** | **.50** |

*Note*. **p* < .05, ** *p* < .01
